# Quantifying resilience potentials in construction: pilot evaluation of the resilience assessment grid

**DOI:** 10.3389/fpubh.2025.1675086

**Published:** 2025-11-12

**Authors:** José Marcelo Tierra-Arévalo, María Carmen Pardo-Ferreira, Pedro M. Arezes, Juan Carlos Rubio-Romero

**Affiliations:** 1 Escuela de Ingenierías Industriales, Universidad de Málaga, Málaga, Spain; 2 Escuela de Ingenieria, University of Minho, Guimarães, Portugal

**Keywords:** construction, potentials, resilience assessment grid, safety II, tool

## Abstract

**Introduction:**

The construction sector’s entrenched hazards and enduring accident statistics necessitate a paradigm realignment - from rigid, checklist-driven Safety-I models toward a dynamic, resilience-centered Occupational Health and Safety Management (OHSM) ethos. This study endeavored to architect a 36-item questionnaire - rooted in Resilience Assessment Grid (RAG) and integrated within the Occupational Health and Safety Plan (OHSP) - to interrogate and quantify the sector’s resilience capabilities.

**Methods:**

Adopting a five-phase development trajectory, commenced with an RAG-aligned draft, secured content validity via the Individual Aggregate Method, iteratively refined items, achieved expert consensus through a multi-round Delphi panel and conducted a pilot implementation on an active construction site. Reliability metrics (Cronbach’s *α* = 0.914) and user acceptability were appraised using a six-point Likert continuum.

**Results:**

The instrument exhibited robust psychometric properties and operational viability. Empirical findings revealed a provisional “sometimes” alignment with resilient performance across the four RAG pillars - Respond, Monitor, Learn and Anticipate. Spider-diagram visualizations translated complex data into intuitive insights, pinpointing focal areas for resilience enhancement.

**Conclusion:**

By transcending conventional audit paradigms, this RAG-based questionnaire delivers a rigorous, actionable blueprint for embedding adaptive capacities within the OHSP. It empowers industry stakeholders and regulators to transition from reactive safety conventions to a proactive, foresight-driven Safety-II framework, fundamentally advancing OHSM in construction.

## 1 Introduction

Bridging the divide between theoretical paradigms and their operationalization in the construction industry is indispensable. When contrasting planned versus actualized work, the critical role of safety management in contemporary construction projects becomes starkly apparent. Notably, the United States, despite representing under 4% of the global construction workforce, accounts for approximately 20% of sector fatalities (1, 2), while Europe similarly registers around 20% of fatal construction accidents (3). Moreover, the construction sector exhibits more than double the accident rate of other industries; falls from height account for 35% of fatal incidents, and 28% result from incompatible simultaneous tasks (4).

In 2022, the European Union (EU) recorded 2.97 million non-fatal occupational accidents, alongside 3,286 fatal incidents. The incidence of fatal accidents across the EU was 1.66 per 100,000 employed people, although this rate varied considerably among Member States. For instance, in 2022 the lowest rates - fewer than 1.00 fatal accidents per 100,000 workers - were observed in the Netherlands, Greece, Germany, Sweden, and Ireland, whereas Bulgaria, France, and Malta exceeded 3.00 per 100,000. Non-fatal accidents were far more frequent, averaging 1,506 per 100,000 workers, with Romania and Bulgaria reporting fewer than 100, in stark contrast to Spain, Portugal, France, and Denmark, where rates surpassed 2000. Sectoral analysis revealed that construction, transportation and storage, manufacturing, and agriculture, forestry, and fishing together accounted for 65.6% of fatal and 43.0% of non-fatal occupational accidents, with construction alone responsible for 22.9% of all fatal cases. Standardized non-fatal accident rates further illustrate national disparities: 3160 in Portugal, 2,733 in France, and 2,706 per 100,000 in Spain. In Spain specifically, accident trends remain concerning: during 2024, the overall number of accidents leading to sick leave across all economic activities increased by 0.1% compared with 2023. Within construction, 81,697 workday accidents were reported in 2024, including 135 fatalities, compared to 83,966 total cases and 131 fatalities in 2023, representing a 3.1% rise in mortality (5).

These data highlight the need for a comprehensive Occupational Health and Safety Management (OHSM) framework to address the complex risks in construction - a Complex Socio-Technical System (CSS) (6). Managers, safety professionals, and workers must balance procedures with adaptive on-site decisions (7). Resilience Engineering (RE), developed over two decades, shifts focus from failures to daily successes under pressure through its Safety-II model (8, 9). Yet construction remains rooted in Safety-I, limiting outcomes. Adopting RE principles could markedly improve construction safety (7). New tools and metrics are needed to build resilience and prevent accidents in construction (10, 11). Yet few studies apply RE in this sector, making its uptake a key challenge (12, 13). We bridge that gap by embedding RE and Safety-II principles into construction safety management.

EU Directive 92/57/EEC established basic safety requirements for temporary and mobile sites (14). Spain adopted these via Royal Decree 1627/1997, which mandates a Health and Safety Coordinator, a Health and Safety Study, and an Occupational Health and Safety Plan (OHSP) for each project (15). Article 7.3 of Royal Decree 1627/1997 mandates a project-specific OHSP - approved by the Health and Safety Coordinator and derived from the foundational study - that aligns planning, organization, and control of site work with safety needs. It is the go-to guide for contractors, subcontractors, and self-employed workers and must be updated when methods or site conditions change, since it details risks, preventive measures, and work phases (16). Generic checklists, by contrast, lack the necessary detail, are seldom shared, and rarely used, eroding safety culture (17). Improving OHSP content and its real-time application is vital for daily OHSM, as strong OHSM implementation boosts safety performance (18). A practical, resilience-centered tool rooted in RE principles can enhance OHSP effectiveness by embedding and measuring resilience capabilities within OHSM frameworks (19).

### 1.1 Resilience engineering support

**Figure 1:**
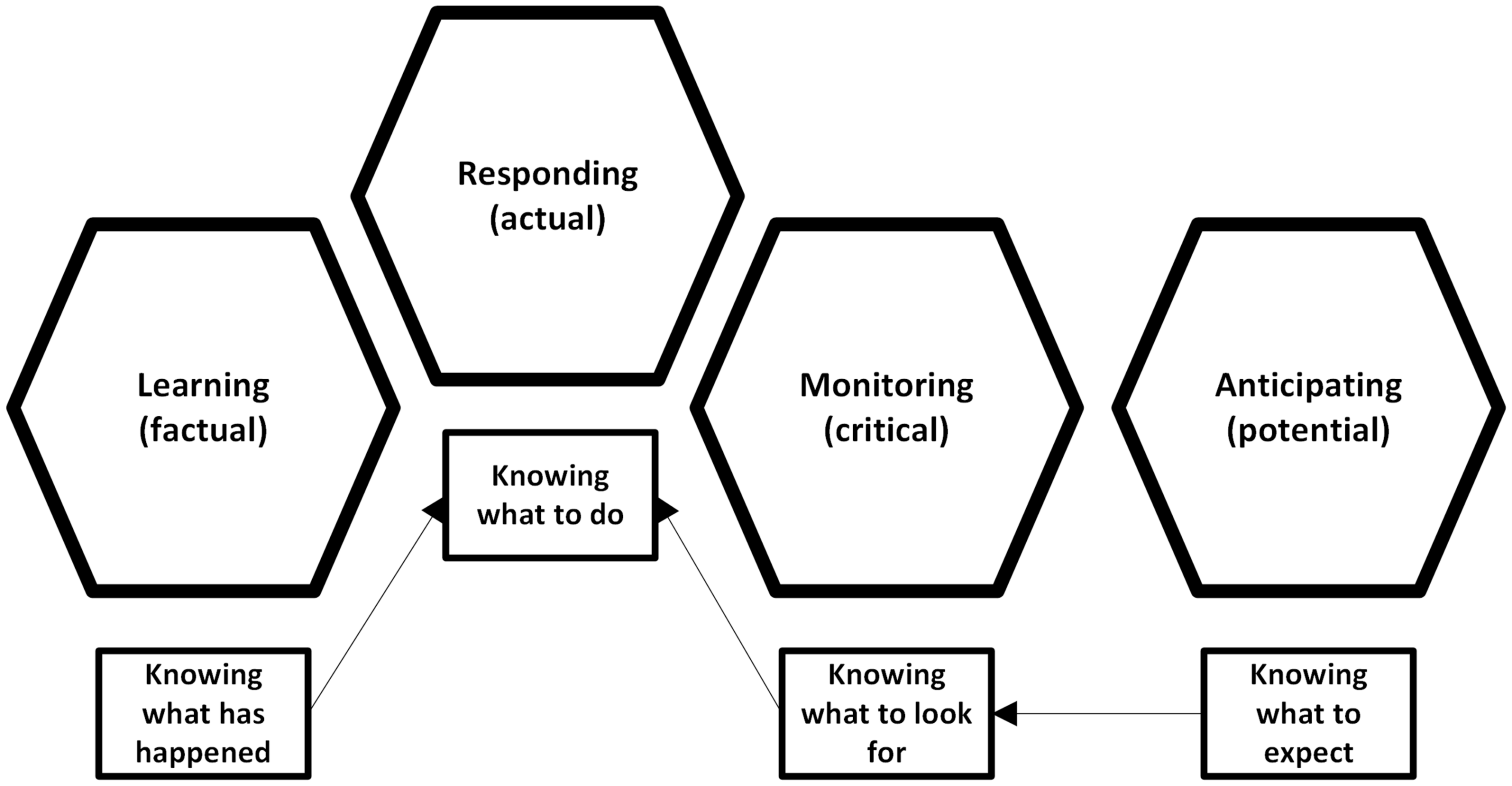
Resilience potentials. Source: adapted from Hollnagel (28).

RE in CSSs describes an organization’s capacity to endure crises, adapt on the fly, and bolster its core capabilities (20, 21). This adaptability often comes from frontline teams’ self-organization and rapid, context-specific actions (22). The Safety-II paradigm, central to RE, shifts focus from failures to how everyday work succeeds through variability management and functional resonance (23, 24). Learning under RE arises through routine operations, structured reflection, and analysis of unexpected events (25). The four resilience potentials - respond, monitor, learn, and anticipate - form the basis for performance surveys (Figure 1), guiding indicator selection to gauge systemic resilience (26, 27). By intentionally developing these capacities, organizations strengthen their ability to absorb disruptions, sustain functions under pressure, and adapt to new demands (8, 28).

Organizational resilience transforms safety management from reactive responses to proactive strategies by clarifying system boundaries, handling disruptions, and speeding recovery (29). Although resilience is now seen as a comprehensive concept needing thorough modeling, the construction field lacks practical tools (26). Agile, on-site methods are vital for applying RE principles and adopting the Safety-II mindset (30). Healthcare’s shift from preventing failures to managing trade-offs under uncertainty highlights this need (31). Promisingly, construction research is using RE methods: the Functional Resonance Analysis Method (FRAM) maps variability (32) and the Resilience Assessment Grid (RAG) - launched in 2010 and refined by 2017 - measures the four resilience potentials within Safety-II frameworks (8, 28, 33). These approaches lay the foundation for resilience-centered safety management tailored to construction.

### 1.2 Resilience assessment grid as a tool

Resilience is four enacted capabilities - respond, monitor, learn, and anticipate - rather than a static trait (27, 34). RAG converts these into a structured questionnaire, yielding tailored indicators to assess resilient performance (26). Since each CSS is unique, RAG deployment must reflect two-way interactions between potentials and context (8, 28, 33). Practitioners collect data through surveys, interviews, and focus groups, reinforcing safety culture (7). Evaluating these four potentials provides a practical proxy for overall resilience and addresses a key empirical gap (35).

Applying RAG across sectors revealed two core benefits (36): First, RAG maps system strengths and weaknesses, guiding tailored resilience profiles that inform adaptive improvements. Second, while novel, RAG offers practical insights into daily CSS operations by measuring potential resilience - rather than resilience itself - and, alone or combined with other methods, provides a solid framework to quantify organizational resilience. Following the Introduction, the paper comprises four sections. Section 2 reviews the background, research gap and aim. Section 3 details the five-stage methodology. Section 4 presents the questionnaire design’s results and discussion. Section 5 summarizes key findings, acknowledges limitations and outlines prospects for future research.

## 2 Background, research gap and aim

### 2.1 Background

A review of RAG deployments over the last decade reveals two principal application modes: as a standalone instrument and in combination with complementary methods. These approaches have elucidated RAG’s versatility in capturing resilience potentials across varied domains.

#### 2.1.1 Standalone applications

Early implementations focused on determining organizational resilience in critical infrastructures, with bespoke RAG-based tools developed for the oil and railway industries (37, 38). More recently, standalone RAG has been employed to:

- Gauge resilient performance in healthcare organizations subjected to Lean interventions (39).
- Support safety management within rail transport (40).
- Address variability and operational demands in the broader transport sector (41).
- Derive resilience indicators for water utilities (42).
- Identify systemic strengths and weaknesses in healthcare settings (43, 44).
- Analyze maritime safety resilience (45).
- Reveal how complexity impedes construction sector Safety Performance Measurement Systems (SPMS) (46).
- Inform the integration of Safety-II practices into daily healthcare routines (47).
- Enhance pandemic preparedness in aviation (48).
- Correlate resilience potentials with quality metrics in healthcare (49).
- Understand and evaluate the resilient performance of a public hospital’s internal medicine department (50).
- Establish the level of resilience capacity in safety management systems within the construction sector (51).
- Assess construction safety-management capabilities under an RE framework (52).

#### 2.1.2 Integrated applications

To enrich resilience profiling, RAG has been combined with:

- Critical Incident Technique (CIT) and Critical Decision Method (CDM) for healthcare resilience evaluation (53).
- Analytic Hierarchy Process (AHP) to prioritize resilience criteria in healthcare (54).
- State Assessment Tool (SAT) to reinforce air-traffic safety management (55).
- Functional Resonance Analysis Method (FRAM) to model adaptive dynamics in aviation (35).
- Analytic Hierarchy Process (AHP) to investigate systemic adaptive dynamics in healthcare (56).
- Technical, Organizational and Environmental (TOE) framework to diagnose construction-sector Safety Performance Measurement Systems (57).
- System Theoretical Accident Model and Processing (STAMP) alongside FRAM for systemic accident analysis in submarines (58)
- FRAM-based studies to deepen understanding of CSS dynamism (59).
- FRAM to capture functional variability in a nuclear-powered submarine system (60).
- Rasch method to quantify resilience potential in organizations leveraging digital technologies to enhance workplace safety and resilience (61).

These diverse applications underscore RAG’s adaptability but also highlight the need for a construction-specific RAG toolkit - one that aligns with RE and Safety-II principles to address the industry’s unique socio-technical complexities.

### 2.2 Research gap

Despite construction’s persistently high accident rates, resilience-focused research remains limited: of 23 RAG-related publications, only four address construction (∼17%) - three independent RAG studies (46, 51, 52) and one combined with the TOE framework (57). No study has yet developed an OHSP-centered RAG questionnaire to assess RE potentials in construction, leaving a critical gap in both OHSM research and practical safety planning. Existing RAG applications offer diagnostic insights but fall short of providing on-site tools for integrating RE in CSSs (26). Bridging this divide requires a tool that converts a decade of theoretical advances into a testable OHSP: piloted on active projects to reveal planning-versus-practice gaps; guiding targeted OHSP revisions to reflect Safety-II realities; and validating RAG’s contributions through documented, iterative improvements, as demonstrated in healthcare (39). Embedding RAG within mandatory OHSPs can transform them into documents that systematically capture adaptations and mirror operational complexity.

### 2.3 Aim and research questions

This study endeavors to develop and validate a RAG - an informed questionnaire, anchored in the OHSP, to systematically appraise the four core RE potentials - Respond, Monitor, Learn and Anticipate - within construction operations.

#### 2.3.1 Research questions

1. By what methodological pathway can an OHSP-centric, RAG-derived instrument be conceived to guarantee conceptual fidelity, content validity and psychometric reliability in measuring resilience capacities?
2. Which pilot testing procedures will most effectively demonstrate the tool’s operational feasibility, diagnostic precision and clarity of result visualization?

#### 2.3.2 Principal contributions

1. A rigorous five-stage development protocol offering a transferable blueprint for resilience assessment across sectors.
2. A validated questionnaire tailored to the construction domain, achieving robust internal consistency.
3. Empirical evidence from an active construction-site pilot, evidencing the instrument’s capacity to identify resilience strengths and deficiencies.

## 3 Research method

**Figure 2:**
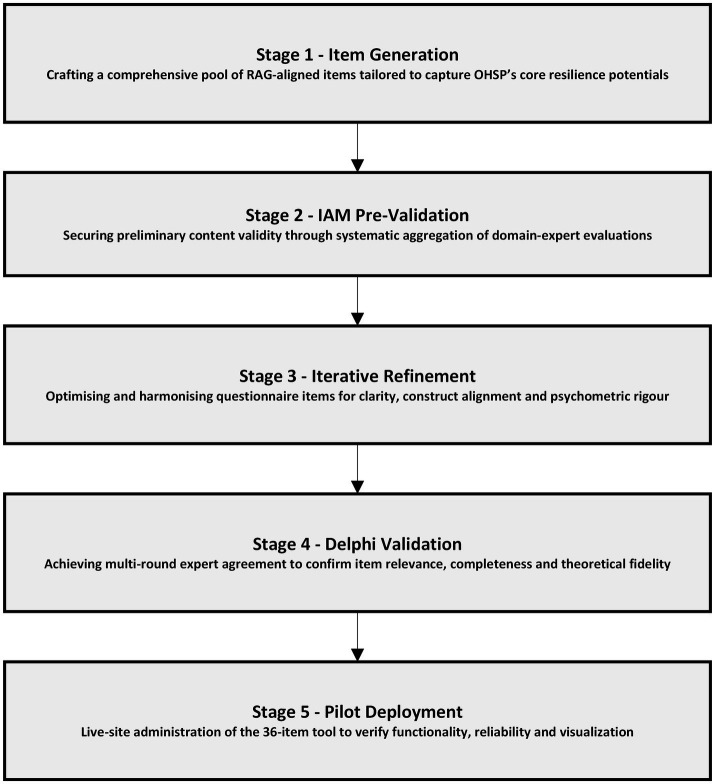
Five-stage empirical framework for developing, validating and pilot testing the RAG-based OHSP resilience assessment tool. Source: authors’ own elaboration.

This study utilizes the RAG framework to measure organizational performance through the four resilience potentials - respond, monitor, learn and anticipate (62). The questionnaire’s structure draws on adaptations of analogous RAG applications in other sectors (41, 42, 56, 59). Given the absence of a Spanish-context RAG questionnaire, the Individual Aggregate Method (IAM) was incorporated in stage 2 to ensure item suitability (63). The methodology comprises five sequential stages (Figure 2).

### 3.1 The framework for designing, validating and testing the RAG-based tool

- Item Generation: A proportional set of items was drafted for each potential, referencing existing RAG questionnaires in oil, rail and other sectors (38, 62) and cross-checked against Hollnagel’s prescriptions (8, 28, 33). Domain expertise informed initial wording.
- IAM Pre-Validation: To address RE’s conceptual novelty and limited Spanish uptake, proposed items underwent IAM evaluation to assess clarity, relevance and phrasing (63).
- Iterative Refinement: IAM feedback guided item refinement, balancing readability and conceptual fidelity to the four resilience potentials.
- Delphi Validation: A panel of construction-safety and RE experts iteratively reviewed and rated items via Delphi rounds, culminating in a consensus-based, validated questionnaire.
- Pilot Deployment: The final instrument was deployed in a live construction firm to assess applicability, functionality and to calculate reliability coefficients. The pilot emphasized tool robustness over result generalization.

### 3.2 Instantiation of the framework

The initial phase entailed drafting items directly from Hollnagel’s “examples of detailed issues” for each resilience potential (8). Supplementary questions were adapted from RAG instruments previously applied in oil, rail and other sectors (38, 62). Four specialists in Occupational Health and Safety, construction and RE reviewed the item pool across three successive iterations, refining wording and alignment to each potential, and thereby producing a first-draft questionnaire. We convened our expert panel using purposive sampling of professional associations, published authors, and peer referrals, under clear inclusion criteria. We balanced representation across disciplines, geographies, and sectors - academia, regulatory agencies, and industry to reduce selection bias. Anonymity was upheld throughout to prevent any single voice from dominating, adhering to established Delphi norms. The questionnaire was pilot tested to refine phrasing and ensure mutual understanding, limiting measurement bias. To maintain engagement, we outlined the time commitment in advance, provided timely reminders, restricted the process to two or three rounds, and kept each survey brief. All consensus benchmarks and feedback mechanisms were clearly defined before the first round.

**Table 2:** Rigorous Delphi panel expert selection criteria.

Criterion	Expertise requirement
1	Authorship - Lead or contributing author on at least three peer-reviewed journal articles in construction safety, RE or related fields.
2	Invited Speaker - Formal invitation to present plenary or keynote lectures at recognized academic or industry conferences.
3	Committee Leadership - Membership or chairmanship of a national-level professional or standards committee in construction safety or occupational health.
4	Industry Tenure - Minimum of 5 years of professional experience within the construction sector, demonstrating applied expertise in safety management.
5	Academic Appointment - Current faculty position at an accredited university, teaching or researching topics aligned with occupational health, safety or resilience.
6	Scholarly Contribution - Authorship or editorial responsibility for books or book chapters on themes pertinent to the study’s subject matter.
7	Advanced Qualification - Possession of at least a bachelor’s degree in civil engineering, architecture or a closely related discipline; postgraduate credentials preferred.
8	Professional Accreditation - Active licensure or certification from a recognized professional body (e.g., chartered engineer, registered safety practitioner).

Source: adapted from: Hallowell et al. (69).

**Table 3:** Characteristics of the panel of experts participating in the Delphi method.

Expert ID	Domain of Expertise	Professional Sector	Years of experience	Academic qualifications	Additional Training
1	Public Administration	Construction and POR	35	Architecture	STORP and HSC
2	Company	Construction and POR	24	Architecture	STORP and HSC
3	University and Company	Construction, installations, and projects	20	PhD in Industrial Engineering	STORP
4	University	Engineering projects and integrated project management	15	PhD in Industrial Engineering	STORP
5	Public Company	POR and environment	18	Industrial Engineering	STORP and HSC
6	Public Administration and Company	POR, Construction, Safety and Health coordination	19	Industrial Engineering	STORP and HSC
7	University	POR, Construction, and Management systems	10	PhD in Industrial Engineering	STORP and HSC
8	University	POR and Management in the construction sector	10	PhD in Architecture	STORP and HSC
9	University	Construction and POR	20	PhD in Architecture	STORP and HSC
10	University and Company	OHS, Construction and Expertise	20	Labor Sciences	STORP
11	Public Company	POR and Environment	15	Chemical Engineering	STORP
12	University, Public Administration and Company	POR, Engineering projects, Safety	36	PhD in Industrial Engineering	STORP
13	Company	Construction and POR	17	Chemical Engineering	STORP and HSC
14	Company	Construction and POR	12	Chemical Engineering	STORP and HSC
15	University and Company	POR, Construction and Health and Safety Coordination	15	PhD in Industrial Engineering	STORP and HSC
16	University and Company	POR, installations, and construction	23	PhD in Industrial Engineering	STORP and HSC
17	Public Administration	POR, Civil protection, and Environment	24	Industrial Engineering	STORP

Source: authors’ own elaboration.

An independent panel of three PhD-qualified experts - each with over a decade of construction-sector experience and working knowledge of RE - evaluated each item’s coherence, relevance, clarity and the overall sufficiency of item sets for each resilience dimension (64, 65). They rated items on a four-point Likert scale. Table 1 defines these evaluation variables.

**Table 1:** Operational definitions of content-validity constructs via the individual aggregate method.

Content-validity construct	Operational definition
Sufficiency	The collective set of items within a given dimension fully delineates the construct’s domain; evaluation emphasizes aggregate item coverage rather than individual item adequacy.
Clarity	Items are articulated with linguistic precision and semantic transparency, ensuring respondents can interpret questions unambiguously.
Coherence	Each item exhibits logical and conceptual congruence with its designated dimension or indicator, safeguarding construct alignment.
Relevance	Items embody the construct’s essential attributes, possessing intrinsic significance that warrants their inclusion in the instrument.

Source: Escobar-Pérez et al. (65).

IAM analysis criteria (64):

- Include items scoring 3–4 from all experts.
- Exclude items scoring 1–2 from all experts.
- Revise items with mixed ratings (one expert scoring 1–2), rewording or deletion as needed.

For the sufficiency dimension - assessing sets of items rather than individual questions - the same thresholds guided whether the collective item set adequately represented each resilience potential.

Building on the IAM findings, the four original item-development experts reconvened to refine the questionnaire. Each item was revisited in light of expert scores and comments: wording was adjusted for precision, alignment with its designated resilience potential was enhanced, and superfluous or ambiguous items were excised. This iterative process yielded a more concise and semantically robust instrument that better reflected the four resilience dimensions.

To achieve consensus on item relevance, the refined questionnaire underwent a Delphi-based validation. Originating at the Rand Corporation in the 1950s, the Delphi Method systematically aggregates expert judgements to inform decision-making (66). Drawing on best-practice guidelines (67–69), we established eight panelist-selection criteria (Table 2) and required each participant to satisfy at least four.

Literature suggests Delphi panels ranging from 8 to 17 experts (69–75). To maximize rigor and validity, we convened 17 specialists. Table 3 summarizes their credentials - namely, Senior Technician in Occupational Risk Prevention (STORP), Health and Safety Coordinator (HSC), Prevention-of-Occupational-Risks (POR) sector affiliation and relevant experience.

Experts rated each item’s importance on a five-point Likert scale. We quantified consensus using mean absolute deviation, following Hallowell and Gambatese’s recommendation, thereby identifying items with strong agreement for retention in the final questionnaire.

The final phase involved deploying the validated RAG-based questionnaire in mid-2022 at a Málaga construction firm responsible for 135 dwellings. The project’s material execution budget was €11,544,691, with a Health and Safety allocation of €160,580, over a 20-month schedule and an average workforce of 65. Thirteen participants were selected by the site’s Health and Safety Coordinator via convenience sampling to minimize operational disruption. The cohort comprised one Section Manager, a Director of Risk Prevention and 11 Senior Technicians in Occupational Risk Prevention.

Our pilot study prioritizes feasibility checks and instrument validation before exploring efficacy. Its main aim is to test the questionnaire’s real-world usability and initial reliability, not to produce broad generalizations. Methodological standards recommend 10–15 participants to trial procedures and spot possible flaws (76) and some pilots have worked with as few as eight participants in simulated settings (61). Focusing on internal validity in a controlled environment bolsters the tool’s reliability and curbs biases from small samples. We will closely monitor the pilot, refine item wording, and adjust data-collection methods to ensure clarity and consistency. Detailed documentation of all changes will support transparency and reproducibility. Validating the instrument’s core properties in this phase will establish the scientific basis for a larger, multi-site study to confirm external validity across varied sites and populations, mirroring research with cohorts of 31, 87, and 144 participants (50–52).

An opening briefing introduced core RE concepts and the survey’s objectives. Participants’ queries were addressed before obtaining informed consent. Completion of the questionnaire required 20–30 min, followed by a 15-min debrief to capture immediate reflections. All responses were entered into Microsoft Excel for item-level analysis, and SPSS v25 was used to compute Cronbach’s alpha for reliability assessment. It is important to note that convenience sampling - non-probabilistic and based on participant availability - limits statistical generalizability. Nevertheless, this pragmatic approach maximized engagement under the site’s scheduling constraints (77).

### 3.3 Evaluation of the framework

Consensus was defined following Hallowell and Gambatese’s criterion, whereby mean absolute deviation must fall below one-tenth of the response scale’s range (69). For our five-point Likert validation, this translated into a threshold of <0.5. In each Delphi iteration, experts received feedback comprising the aggregated median and absolute deviation for every item, alongside dedicated comment fields. To optimize data collection and analysis, the questionnaire was deployed via Lime Survey v1.92. Upon conclusion of the Delphi rounds, the finalized 36-item instrument was confirmed (Appendix A). For empirical application, the refined questionnaire was administered to construction-site personnel. In accordance with Hollnagel’s guidance, respondents rated each item’s occurrence on a six-point frequency scale - from “Never” (0) to “Always” (5) - thereby aligning measurement with RE practice (78).

To evaluate the OHSP’s contribution to resilient performance, the final 36-item RAG questionnaire was completed by 13 site participants. Responses were categorized by the four resilience potentials and scored on a six-point Likert scale. Weighted means for each item and an overall mean per potential were calculated to quantify the organization’s resilience profile (8, 79). Results were plotted on a radar chart to illustrate each potential alongside the aggregate resilience score. Finally, Cronbach’s alpha was calculated in SPSS v25 to assess internal consistency. The obtained coefficient exceeded the conventional 0.7 threshold, attesting to the tool’s reliability (56, 77).

## 4 Results and discussion

This section addresses the research questions outlined previously.

### 4.1 Designing an OHSP-centric RAG questionnaire to assess RE potentials in construction

Stage one commenced with the formulation of 68 provisional items, drawn from Hollnagel’s detailed potential-related prompts and sector-specific RAG instruments. These items underwent three consecutive expert review cycles, during which questions were refined, amalgamated or excised in light of specialist feedback. The iterative process yielded a 44-item draft questionnaire, systematically aligned with the four resilience potentials: 10 items probing Learning, 12 addressing Responding, 11 evaluating Monitoring and 11 examining Anticipating (8).

The IAM was employed to validate item content. Sixteen items were identified as problematic, with at least one expert assigning a score of 2. In adherence to our pre-established criteria, any item rated 1 or 2 was slated for revision, rewording or exclusion. These items were thus reviewed in Stage Three.

In Stage three, items flagged via IAM were systematically revised. For the Responding potential, two questions were excised and one rephrased; Monitoring saw one deletion and two edits; Learning required a single wording adjustment; and Anticipating - challenged by the OHSP’s typically shorter project horizon - underwent five removals and four substantive rewrites. The difficulty in crafting anticipation items likely reflects the tension between long-term foresight and finite construction timelines.

The refined questionnaire now comprises 10 items for each of the Responding, Monitoring and Learning, with 6 items dedicated to Anticipating. This configuration aligns with sectoral precedents: healthcare RAG instruments have spanned 32–38 Likert-type items (43, 44), transport systems 29 items (41), and water utilities 16 items (42). At the high end, aviation resilience assessments employed 56 items, validated by 42 industry experts (48). Conversely, some healthcare studies have released preliminary RAG questionnaires pending further validation (39).

Seventeen experts, each fulfilling at least four of the eight selection criteria (Table 2), participated in the Delphi rounds to assess item importance. The process unfolded as follows:

- Three iterative rounds were conducted to refine consensus on each item’s inclusion.
- By Round 2, full agreement (mean absolute deviation < 0.5) was achieved for 34 of the 36 items.
- The remaining two items - one in the Learning potential and one in Monitoring -reached consensus in Round 3.

Due to the prior IAM screening, no item received ratings below the acceptable threshold; consequently, all 36 items were retained in their final form. This rigorous, multistage validation underscores the critical role of expert consensus in tailoring the questionnaire to construction-sector realities (42).

Stage five entailed two principal psychometric assessments. Firstly, validity was examined - the extent to which the instrument measures its intended constructs and yields sound inferences - building on initial content validation via the IAM and subsequent criterion and construct validity appraisals through the Delphi Method. Psychometric theory asserts that overall validity comprises content, criterion and construct facets; accordingly, each facet was evaluated independently to derive a comprehensive validity determination for the full questionnaire. Secondly, after implementing the validated RAG-based questionnaire - whose detailed outcomes appear in the next section - we examined reliability, defined as consistency and coherence. Using the five-point Likert scale, internal consistency was quantified via Cronbach’s alpha in SPSS v25, demonstrating psychometric integrity (56, 77).

Cronbach’s alpha coefficient is a cornerstone metric for assessing a scale’s internal consistency, quantifying the degree to which a suite of items coherently taps a singular latent dimension. This rigorous evaluation is essential in the development and validation of multi-item questionnaires, safeguarding the instrument’s reliability and ensuring each item contributes meaningfully to the construct (77). In our analysis, the alpha coefficient attained a value of 0.914, unequivocally demonstrating the instrument’s robust reliability (56, 77).

#### 4.1.1 Highlights of each potential’s questions

In evaluating the Responding potential, our panel underscored the criticality of an OHSP that is lucid and pragmatically deployable. The literature warns that excessively detailed or opaque safety plans impede real-world responsiveness, undermining resilient performance. Consequently, our instrument probes whether the OHSP: systematically identifies both routine and emergent risks; prescribes calibrated response actions; mandates regular plan updates; allocates resources for peak-demand scenarios; invites iterative refinements from contractors and freelancers to align procedural guidelines with operational realities; integrates robust communication and coordination networks; empowers frontline personnel to enact context-specific adjustments; and undergoes rigorous oversight by the coordinator. As one expert noted, institutionalizing “mandatory briefings on OHSP measures and fostering interactive engagement with contractors, subcontractors and workers regarding planned methods and site organization” is indispensable for cultivating a resilient response ethos.

Our expert panel identified that Monitoring potential demands indicators that are both immediately intelligible and rigorously calibrated to capture site-level performance nuances. This meticulous surveillance is crucial, for the four resilience potentials - Respond, Monitor, Learn and Anticipate - function as an interdependent system; any opacity in real-time operations undermines adaptive responses, iterative learning and strategic foresight. Notably, experts accorded retaliative, reactive indicators moderate value, reaffirming RE’s strategic emphasis on proactive metrics as the bedrock of organizational resilience. Consequently, our instrument probes the establishment of precise monitoring workflows; the development of coherent, valid and reliable indicators; the institutionalization of cyclical indicator audits to uphold relevance; the calibration of measurement cadence; and the guarantee of minimal lag between data capture and actionable insight. One expert encapsulated this imperative: “The definition and deployment of monitoring indicators within the OHSP are a critical linchpin,” while another declared periodic indicator appraisal “fundamental” to sustaining resilient performance. Echoing these insights, it had been demonstrated in a healthcare context that Monitoring was rated lowest among seven expert-evaluated potentials, even as Responding, Learning and Anticipating were deemed satisfactory (47). Their findings advocate for the seamless integration of Safety-II ethos into organizational culture and daily operational routines to engender lasting resilience.

Our expert panel concurred that the efficacy of the Learning potential hinges on unequivocally delineating which site events warrant reporting (80–82). Beyond this, they stressed the imperative of a structured incident inquiry process, codified analytic protocols, expedited knowledge-extraction cycles and the allocation of sufficient resources. Fostering a culture that systematically harvests lessons from both successes and setbacks - via scheduled debriefs and a formal feedback mechanism - was deemed essential. One expert aptly remarked, “learning to respond is the basis of resilience.” This finding resonates with cross-sector RAG literature, where Responding and Learning routinely emerge as dominant potentials (41, 44, 49). Intriguingly, construction-sector studies reveal a shifting hierarchy - Monitoring once topped the list before yielding to a more balanced profile in a RAG-TOE deployment - highlighting the sector’s dynamic complexities (46, 57). Moreover, some researchers pinpoint Learning and Anticipating as the most demanding dimensions to operationalize within construction settings (52).

In the Anticipating potential, our expert panel underscored the imperative that those entrusted with forecasting site-specific vulnerabilities and systemic threats possess deep experiential insight, dedicated analytic capacity and commensurate resources. Complementary survey items probe whether the OHSP explicitly identifies and mitigates emerging weaknesses; whether a formalized, proactive anticipation methodology is in place; and whether feedback loops engage every contractor, subcontractor and site operative. Additionally, the instrument examines the establishment of communication infrastructures for transmitting anticipatory findings and the integration of real-time detection mechanisms that update the OHSP continuously. As one expert aptly stated, “the capacity to anticipate is by far the most formidable.” Illuminating sectoral contrasts, it has been reported that within maritime public enterprises, the hierarchy of resilience potentials places Respond foremost, Learn next and Monitor last - echoing a common RAG archetype - whereas private maritime firms reverse this ordering, elevating Monitor above Respond and relegating Anticipate to the final tier (45). By comparison, another study had achieved similar validation objectives with a concise 16-item scale (37). Drawing on these insights, some researchers argue that bolstering Anticipation and Monitoring demands an enriched comprehension of emergent phenomena arising from robust Learning and Responding cycles, thereby forging a truly adaptive and foresight-driven OHSP (49).

Synthesizing the four resilience potentials, our bespoke RAG-based questionnaire was conceived to embed resilience firmly within the OHSP framework on construction sites (10, 11). One expert astutely observed that “with a forward-looking, practical and efficient OHSP it is difficult to maintain the current criteria” encapsulating the tension between established safety norms and emergent resilience paradigms. Throughout the Delphi process, panel members reiterated that realizing RE’s transformative promise will necessitate a profound strategic shift across the construction sector - and especially within OHSP practice (36). The culmination of this endeavor is a rigorously validated, 36-item instrument designed to measure the OHSP’s contribution to resilient performance and to reinforce OHSM in construction (16). Crucially, as some researchers affirm, the active engagement of sector experts has been pivotal in bridging the conceptual divide between safety planning and safety governance (42).

While existing RAG applications offer insights, they lack on-site tools to integrate RE into construction’s CSSs (26). We addressed this by developing an OHSP-based instrument that operationalizes RAG theory. Testing it on active projects uncovers gaps between planned and actual safety practices, guides targeted OHSP updates in line with modern safety principles, and documents iterative improvements to validate RAG’s impact. Embedding RAG into mandatory OHSPs transforms them into documents that chronicle adaptations and reflect real-world complexity. Under Spanish law, every construction project needs an OHSP - approved by the Health and Safety Coordinator and based on the foundational study - that aligns planning, organization, and control of site work with safety requirements (16). Enhancing OHSP content and its application is vital for effective OHSM, as strong OHSM implementation enhances safety performance (18). By applying the IAM alongside Delphi panels - unlike previous RAG tools - we reinforce the instrument’s validity and ensure its fit for practice.

### 4.2 Pilot implementation of the RAG-based OHSP instrument: validating functionality, efficacy and outcome visualization

During the pilot implementation, 13 construction professionals engaged with the rigorously validated 36-item RAG instrument. Employing a six-point Likert continuum, we calculated weighted means for each resilience potential alongside a composite resilience index (26, 27). The systematically tabulated findings were then translated into a spider diagram, providing an intuitive visual narrative of the organization’s resilience profile (42).

**Figure 3:**
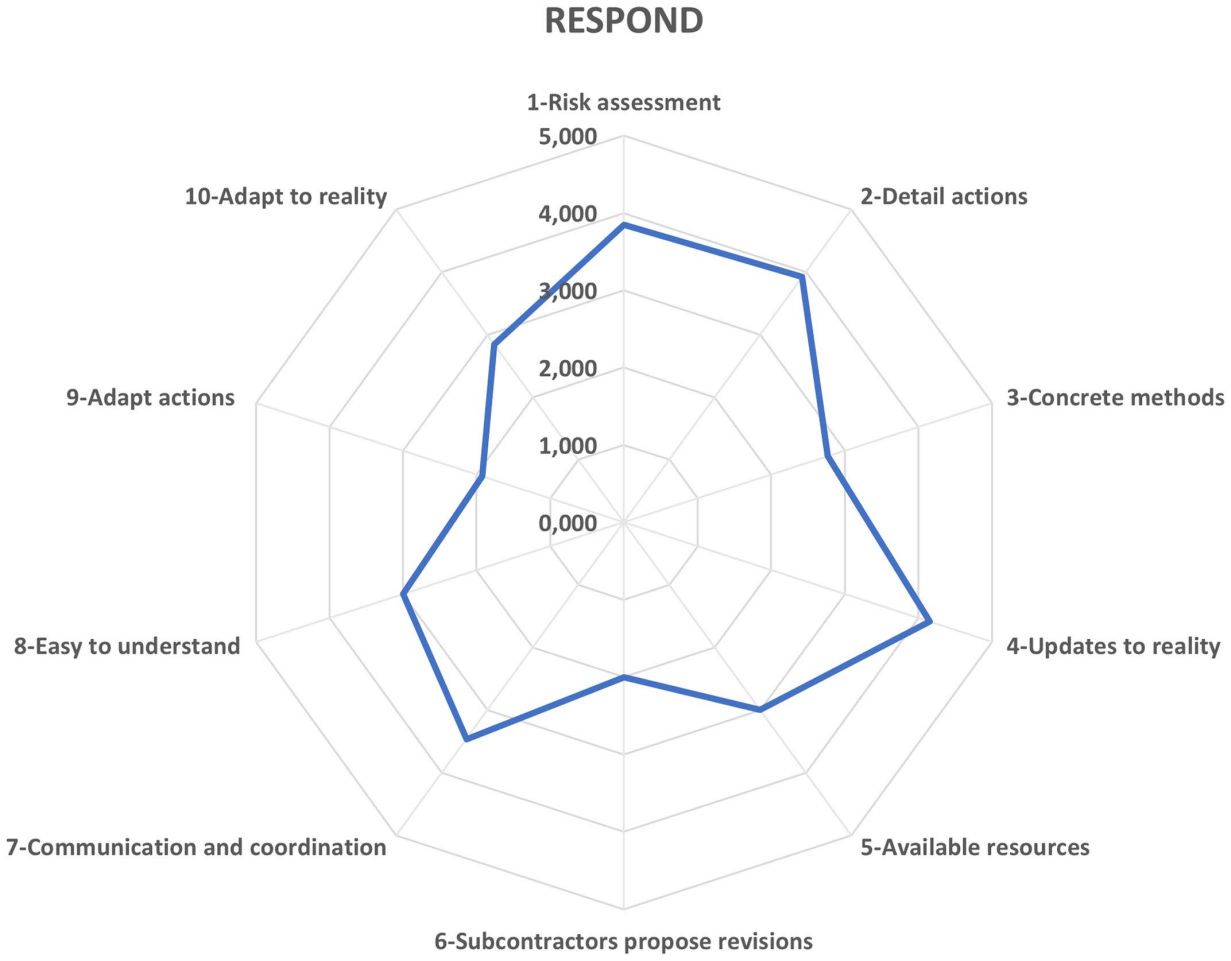
Potential to respond. Source: authors’ own elaboration.

Anchored in the definition of RAG as a metric for core resilience functions (62), Figure 3 charts the Responding potential. A value of 3.09 on the six-point continuum suggests the organization “Often” executes the responsive behaviors essential to resilient construction delivery. Disaggregating this composite score, the OHSP demonstrates strong procedural agility, garnering 4.15 (“Almost always”) for dynamically updating its guidelines in step with operational plan modifications. In stark contrast, it records merely 1.92 (“Almost never”) on empowering site personnel to self-initiated action adjustments - revealing a strategic focus area for bolstering adaptive capacity.

When assessing the Responding potential, safety managers recognize two foundational strengths. First, the OHSP remains tightly aligned with dynamic operational plans. Second, it prescribes robust preventive actions and clear reactive procedures for critical incidents. Yet the plan’s adaptive spirit shows two gaps. It rarely taps into subcontractors’ and independent tradespeople’s practical expertise to enhance its guidance. And it generally does not empower workers to adjust their behaviors independently in response to evolving site conditions.

**Figure 4:**
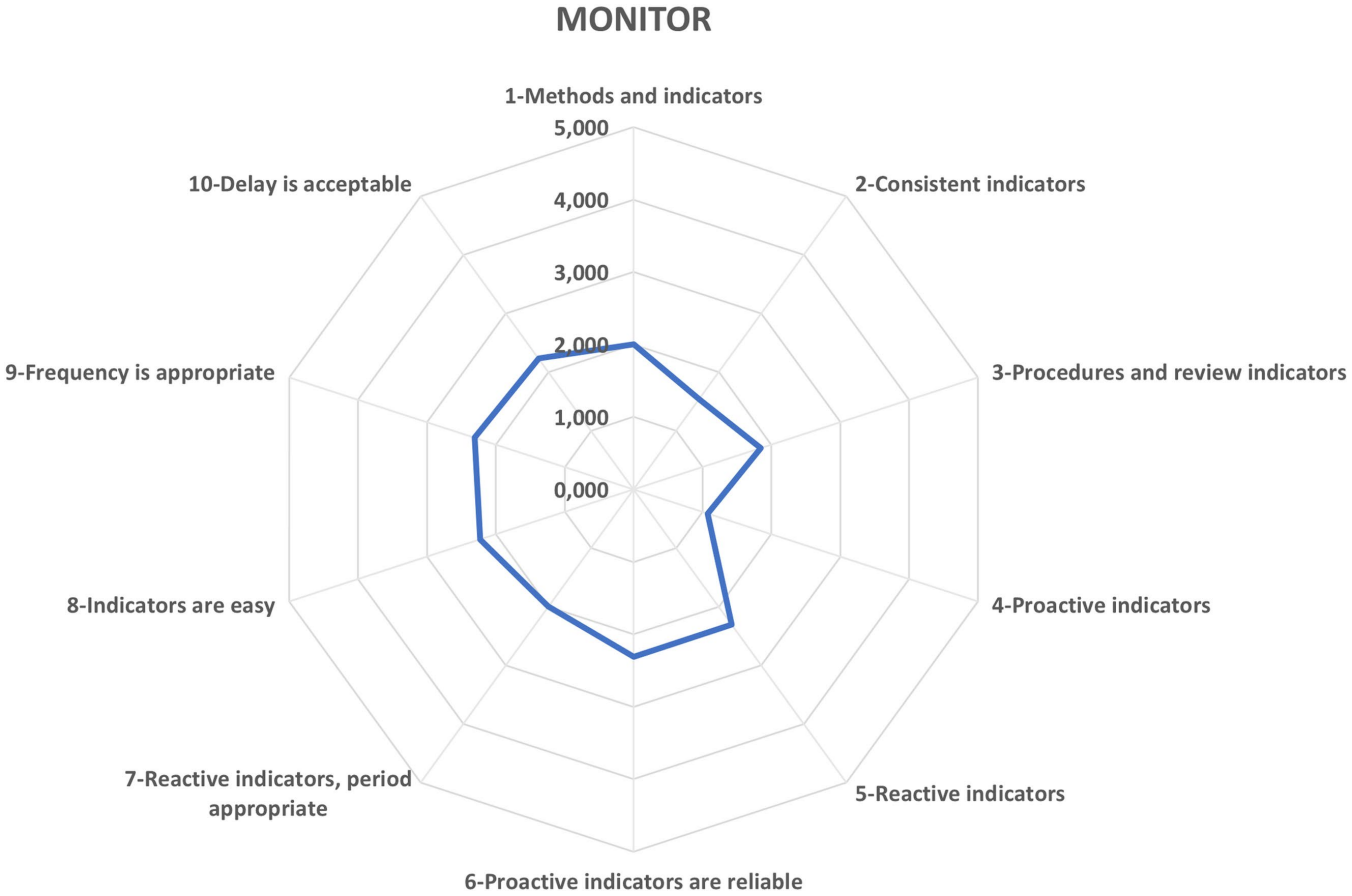
Potential to monitor. Source: authors’ own elaboration.

In Figure 4, the Monitoring potential emerges as a critical vulnerability, with a composite score of 1.99, indicating that the organization “Almost never” achieves the sustained oversight necessary for resilient construction delivery. Disaggregated metrics reveal that the OHSP attains a modest 2.31 (“Sometimes”) for codifying reactive indicator schedules, enforcing the validity and reliability of proactive measures, and calibrating measurement cadences. Alarmingly, it registers a mere 1.07 (“Almost never”) for embedding proactive indicators - a deficiency that undermines anticipatory resilience and demands immediate strategic remediation.

In Monitoring, safety managers see moderate progress: the OHSP tracks reactive indicators (injuries and accidents) and validated proactive metrics at a sensible frequency. Participants agree these measures can be both simple and informative. Yet the plan seldom defines metrics tailored to actual site workflows or routinely includes forward-looking measures like pre-task briefing attendance or counts of safety-promotion efforts. Closing these gaps offers a pivotal opportunity to shift Monitoring from mere procedure to a strategic tool for enhancing resilience in construction.

**Figure 5:**
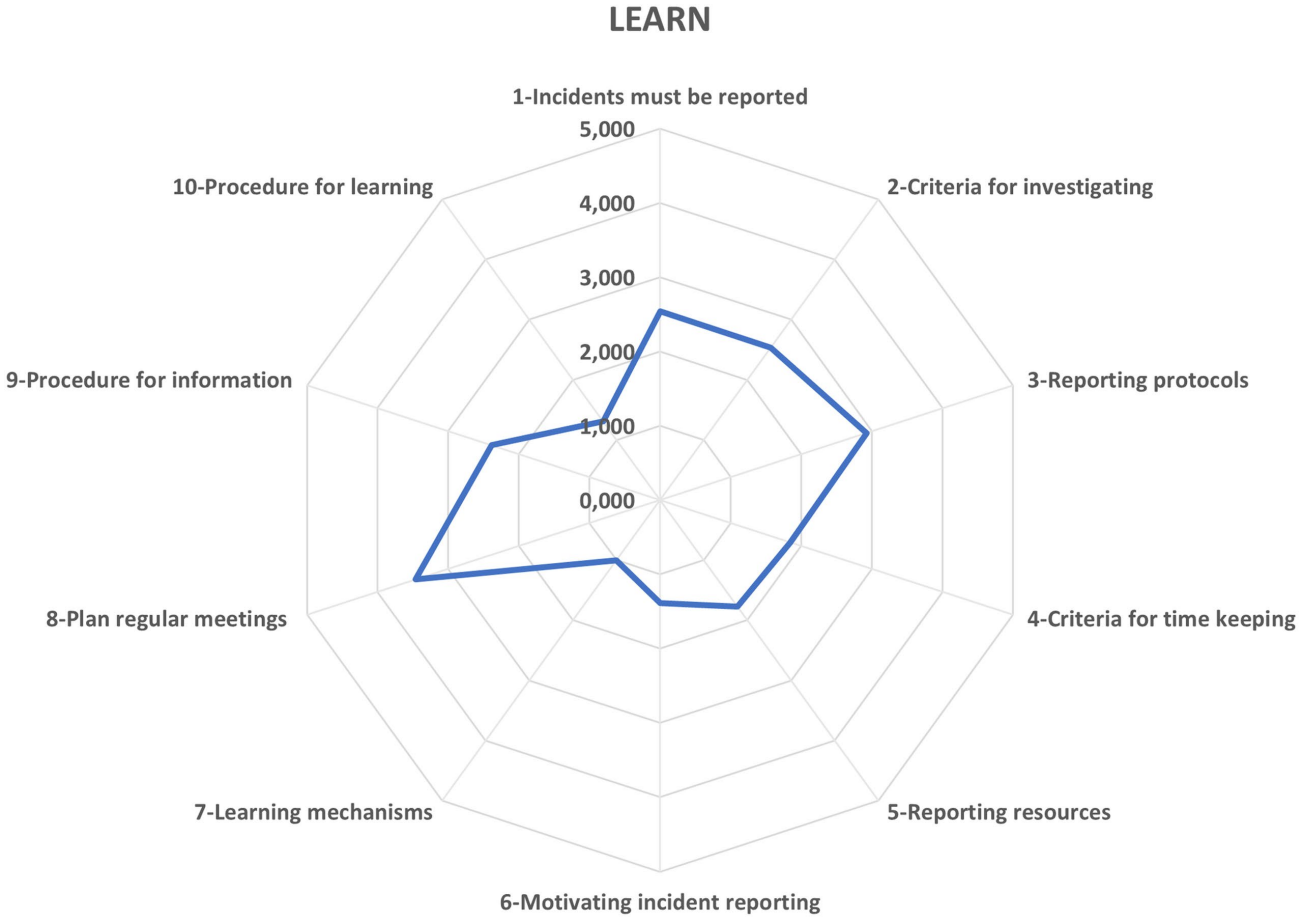
Potential to learn. Source: authors’ own elaboration.

Figure 5 elucidates the Learning potential, registering a value of 2.12, which denotes that the organization intermittently engages in reflective practices essential for resilient construction delivery. Within this profile, the OHSP demonstrates strength - 3.46 (“Often”) - by institutionalizing regular, cross-functional meetings that scrutinize successes as rigorously as failures. Yet, a critical gap emerges: a mere 1.00 (“Almost never”) on embedding formalized mechanisms to harvest insights from favorable outcomes, revealing an untapped avenue for enriching organizational learning and fortifying future resilience.

In the Learning potential, safety leaders observe a mixed picture. The OHSP holds regular, site-wide briefings to review both errors and successes, and it sets clear protocols for sharing unexpected incidents with all teams. These practices foster reflection, but the plan rarely converts insights into new procedures, training, or structural changes. It also does not systematically document lessons from positive outcomes alongside failures. Closing this gap is crucial: by formalizing and applying lessons from each debrief construction sites can shift from occasional reflection to continuous learning and stronger resilience.

**Figure 6:**
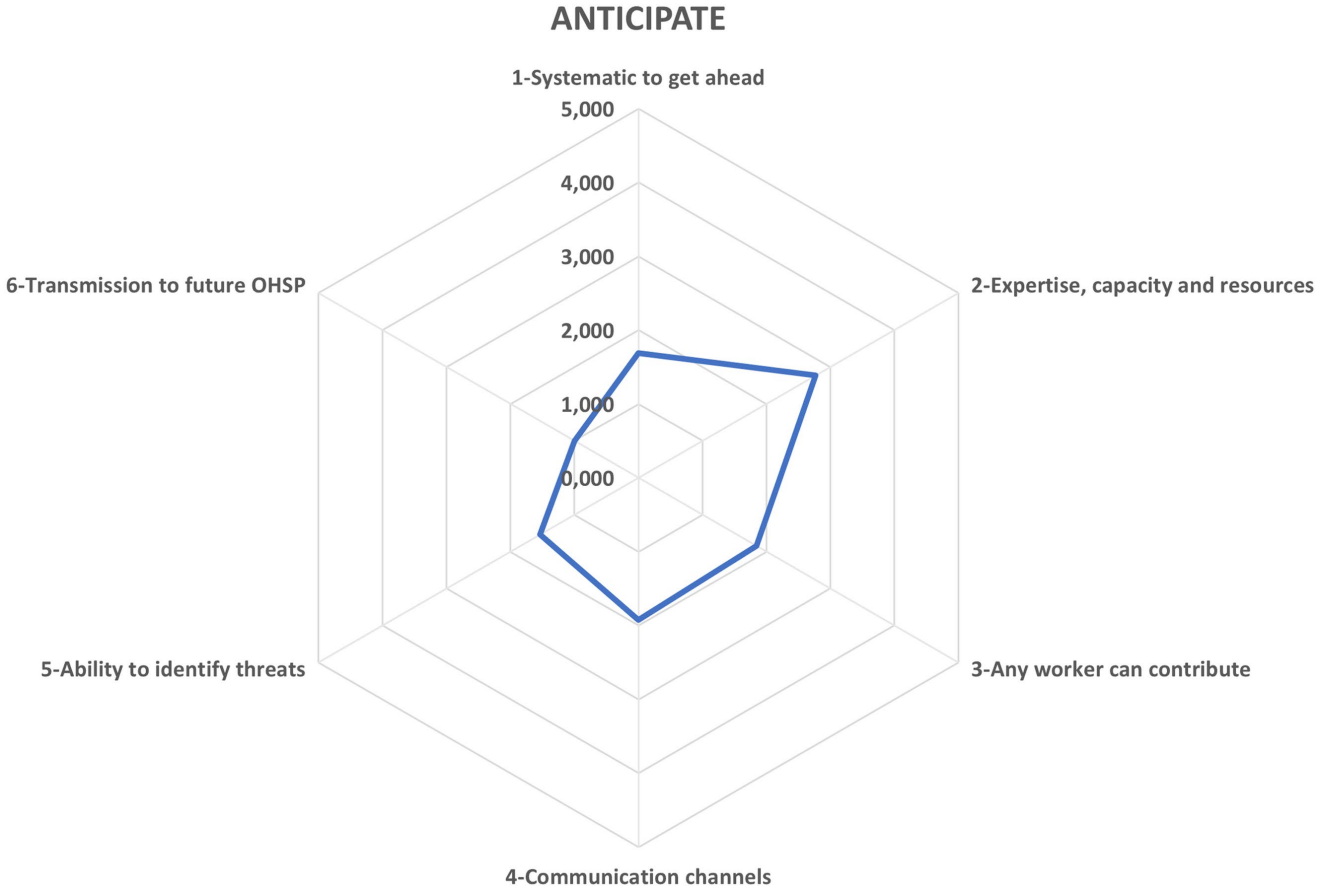
Potential to anticipate. Source: authors’ own elaboration.

In Figure 6, the Anticipating potential emerges as the organization’s weakest link, with a composite score of 1.80 - interpreted as “Almost never” - highlighting a profound gap in proactive foresight crucial for resilient project delivery. Although the OHSP moderately supports this function (2.77, “Sometimes”) by appointing skilled analysts with the requisite capacity and resources, it completely fails (1.00, “Almost never”) to embed formalized feedback loops that ensure identified threats and emerging opportunities inform successive plan iterations. This stark discrepancy underscores the urgent need to institutionalize robust anticipatory mechanisms within construction OHSP frameworks to close the resilience cycle.

For the Anticipation potential, OHS experts point to it as the organization’s key obstacle. While it is promising that practitioners sporadically offer the expertise, capacity, and tools needed to proactively identify threats, the OHSP rarely embeds formal pathways to foster and preserve this foresight. Even more telling is the absence of formal feedback loops: identified threats and emerging opportunities rarely translate into updates of the safety plan. In our Delphi rounds, Anticipation saw the steepest attrition - five of eleven provisional items were removed - resulting in only a 54% retention rate and underscoring the challenge of capturing this nuanced capability. Consequently, across the construction resilience landscape, professionals view Anticipation as the hardest potential to establish, closely followed by Monitoring and Learning.

**Figure 7:**
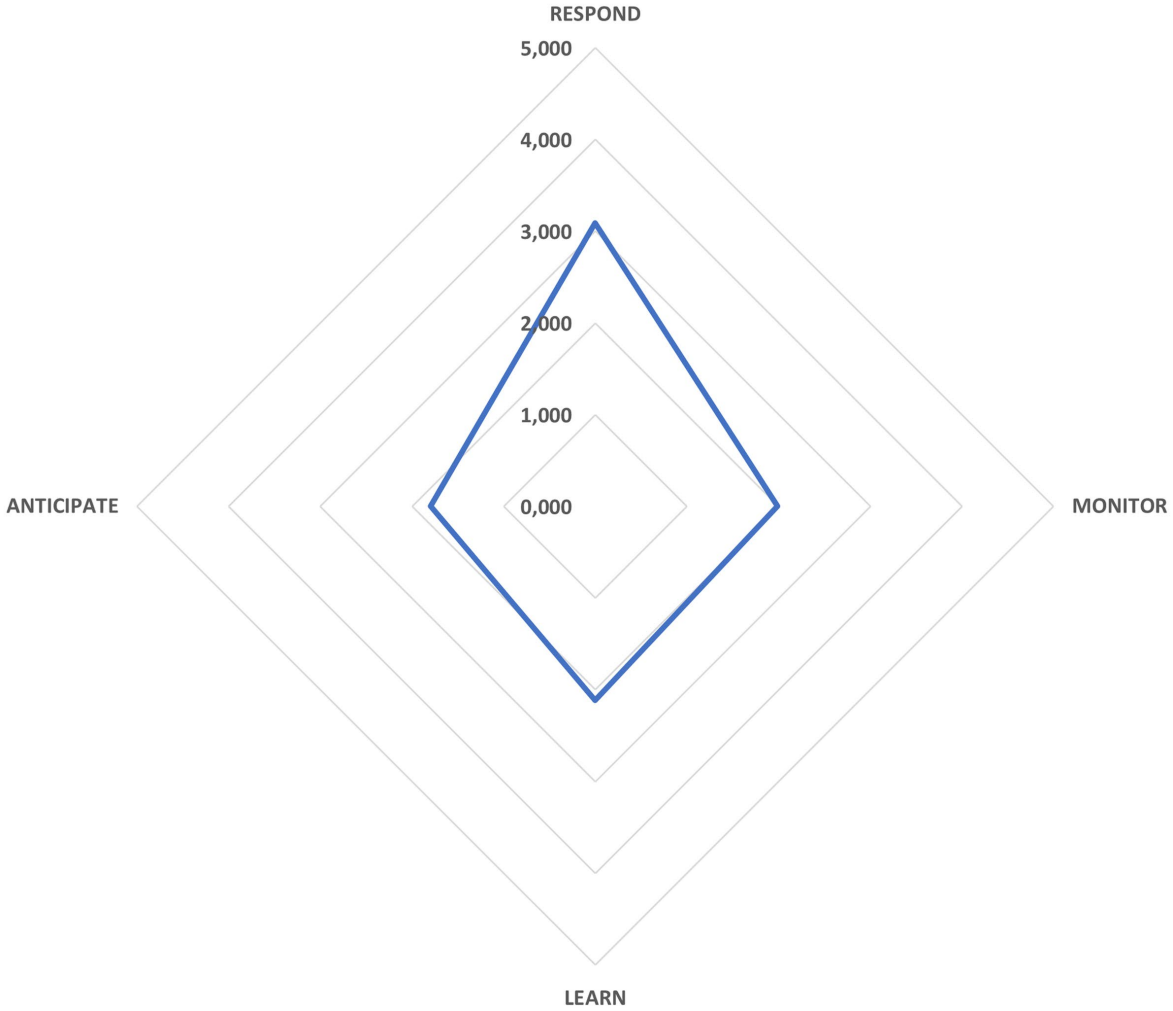
Potentials for resilient organizational performance. Source: authors’ own elaboration.

In Figure 7, the quartet of resilience potentials coalesces into one snapshot, revealing a composite score of 2.25 - interpreted as an intermittent (Sometimes) display of these core skills. While the organization can mobilize its resilience capabilities on occasion, this result underscores the need to elevate performance toward more consistent, “Often” or “Almost always” levels if true construction-site resilience is to be realized.

**Figure 8:**
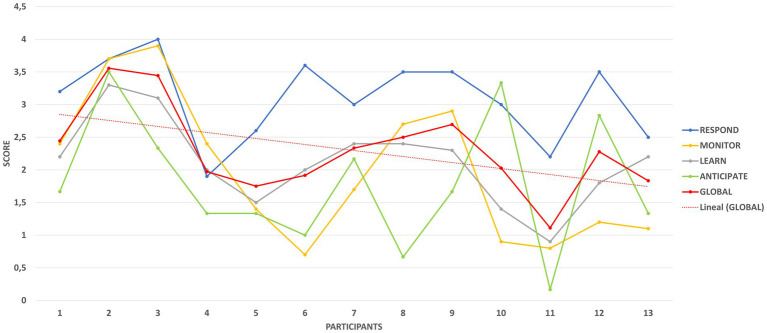
Trend in the assessment of resilient performance in the organization. Source: authors’ own elaboration.

In Figure 8, the descent of the resilience performance curve serves as an early warning: if left unchecked, this incipient decline can snowball into deeper vulnerabilities. This visual insight compels organizations to act swiftly - translating RAG findings into targeted corrective strategies to stop the slide and bolster adaptive strength (7).

## 5 Conclusion

This study culminated in a RAG questionnaire that was pre-validated through the IAM, refined via three Delphi rounds, and confirmed as highly reliable with a Cronbach’s alpha of 0.914. The active involvement of construction-sector experts forged a vital nexus between the OHSP and safety administration. The methodological and substantive lessons gleaned will underpin continuous tuning of questionnaire items and deployment protocols, ensuring the tool remains an adaptive, gauge of on-site resilience within evolving construction contexts.

Envisioning construction through the prism of RE transforms the OHSP from a static compliance document into a blueprint for positive safety and health outcomes. Our data lay bare a sector still mired in Safety-I’s reactive stance - especially in its struggle to anticipate future hazards - suggesting that genuine resilience arises more from frontline improvisation than from formal procedures. This tension underscores a critical imperative: to map, measure and master each of four resilience cornerstones - learning from triumphs and failures, responding to the full gamut of events, monitoring across time horizons, and anticipating what lies ahead.

By treating the construction site as a dynamic sociotechnical network, we can quantify its resilience and spotlight where it falters. The bespoke RAG questionnaire we have developed offers a hands-on, efficient pathway to embed these principles within the OHSP. Yet, crunching the numbers is only half the battle; the real test lies in reconciling prescriptive safety mandates with an adaptive, forward-looking ethos. Our Delphi-driven expert panel was unequivocal: unlocking RE’s game-changing potential demands a wholesale strategic pivot - starting at the very heart of OHSP protocols. In marrying robust psychometric rigor with practitioner wisdom, our instrument not only measures but also bridges the divide between safety planning and agile safety governance, charting a new course for OHSM in construction.

This investigation into Spain’s construction resilience landscape distils critical guideposts for both practitioners and policymakers. Construction enterprises are invited to conduct focused RAG assessments, diagnose underperforming resilience potentials, shore up these vulnerabilities and continuously elevate their performance thresholds. Concurrently, regulators have an unparalleled opportunity to codify RE tenets within national occupational safety regulations, shifting the paradigm from reactive compliance to anticipatory governance. By integrating these standards into procurement criteria, training mandates and inspection protocols, authorities can foster a proactive safety culture poised to eradicate accidents.

Our pilot was not designed for broad extrapolation but to ascertain the questionnaire’s psychometric robustness, operational feasibility and illustrative output design. Constraints inherent to the host organization - most notably reliance on a convenience sample - reflect real-world research challenges. Nevertheless, the validated methodology and instrument offer a transferable template for resilience measurement across varied construction contexts and serve as a springboard for interdisciplinary application. Our pilot and Delphi phases took place under Spain’s specific regulatory and organizational conditions, which can influence hazard focus, wording, and response patterns. To use the instrument elsewhere, a structured cross-cultural adaptation and revalidation process is essential. We also note common Delphi drawbacks - selection bias, similar expertise among panelists, and possible conformity effects. We addressed these through purposive sampling, anonymous rounds, and transparent reporting, yet some bias may remain. Finally, since experts drove item refinement, their viewpoints may have outweighed on-site experience. Integrating cognitive interviews, direct field testing, and a multi-site psychometric study would help balance specialist input with practical insights.

The Anticipation potential demands further scrutiny. We recommend a bifurcated research program: first, methodological refinement to optimize item clarity and relevance; second, rigorous field trials to elucidate the cognitive, organizational and resource-based factors that modulate perceived difficulty. Subsequently, scholarships should unravel the systemic barriers to implementing proactive safety regimes, laying the groundwork for targeted interventions. Finally, longitudinal deployments of the instrument - spanning enterprises of diverse scale and revisited at regular intervals - will illuminate temporal resilience dynamics, validate emergent patterns and catalyze iterative enhancement of both tool and methodology. Accordingly, a larger future investigation is advised - one that systematically fortifies external validity by gathering data from multiple construction sites and a broad range of participants, using samples of at least 30 individuals per setting.

## Data Availability

The original contributions presented in the study are included in the article/Supplementary material, further inquiries can be directed to the corresponding author/s.
