# Supplementary material for: Quantifying resilience potentials in construction: pilot evaluation of the resilience assessment grid
Source: Front Public Health. 2025 Nov 12;13:1675086. doi: 10.3389/fpubh.2025.1675086 (PMC12651450; doi:10.3389/fpubh.2025.1675086)
Supplement: Supplementary file 1 [file Supplementary_file_1.pdf]

APPENDIX A

1 Final questionnaire to assess the contribution of the Occupational Health and Safety Plan (OHSP) to Occupational Health and Safety Management

1.- RESPOND

1 The OHSP identifies and assesses critical risks and events, whether frequent or unforeseen.
2 The OHSP specifies the measures and actions required to prevent these critical risks or events and details the responses to be executed should they occur.
3 The OHSP encompasses the planning of the specific work methods to be implemented in practice.
4 The OHSP is updated in accordance with changes to the planning of operations as actually executed.
5 The OHSP documents available resources (personal, equipment, materials, etc.) to maintain the capacity to carry out work safely under pressure or high workload.
6 Subcontractors and self-employed workers are encouraged to propose revisions to the plan so that it aligns with the resources and methods actually employed.
7 The OHSP outlines communication and coordination systems among contractors, subcontractors, and self-employed workers to facilitate effective work performance.
8 The OHSP is easy to comprehend and is practically applied during work execution.
9 The OHSP allows workers to adjust their actions as deemed appropriate.
10 Prior to approving the OHSP, the Health and Safety Coordinator verifies that the plan's content adequately reflects actual project conditions.

2.- MONITOR

1 The OHSP defines methods and indicators to adequately monitor the execution of work.
2 When safety indicators are defined, they align with the actual work carried out on site.
3 The OHSP establishes procedures and criteria for the regular and appropriate review of indicators, so they remain adapted to reality.
4 The OHSP plans the use of proactive indicators (e.g., percentage of pre-task planning meetings or number of safety-promotion activities).
5 The OHSP plans the use of reactive indicators (e.g., accident or injury rates).
6 The proactive indicators are valid and reliable.
7 The period covered by the reactive indicators is appropriate.
8 The indicators are user-friendly while remaining efficient.
9 The measurement frequency of the indicators is appropriate in the OHSP.
10 The delay between measurement and analysis of indicator results is acceptable.

3.- LEARN

1 The OHSP clearly defines the types of incidents (unexpected or unforeseeable events) that must be reported.
2 The OHSP sets criteria to ensure that information on incidents (unexpected or unforeseeable events) is properly investigated.
3 The OHSP establishes protocols to report these incidents (unexpected or unforeseeable events) to all organizations involved in the project.
4 The OHSP sets criteria to ensure that the time from incident reporting (unexpected or unforeseeable events) to analysis and learning is acceptable.

5 The OHSP defines sufficient resources for the preparation of reports on these incidents (unexpected or unforeseeable events).
6 The OHSP defines mechanisms to encourage employees to report incidents (unexpected or unforeseeable events).
7 The OHSP defines mechanisms for learning from both successful outcomes and failures.
8 The OHSP schedules regular meetings involving all project stakeholders to learn not only from failures but also from successes.
9 The OHSP includes a formal procedure for collecting, classifying, and analyzing all received information and data (reports or indicators, etc.).
10 The OHSP includes a formal procedure for learning from this information (development of new procedures, training, redesign, reorganization, etc.).

4.- ANTICIPATE

1 The OHSP establishes a systematic approach to anticipate potential weaknesses and threats related to safety.
2 Those conducting this analysis of potential weaknesses and threats possess the necessary experience, capabilities, and resources.
3 The OHSP establishes systems to ensure that any worker from the various firms involved in the project can easily contribute information regarding potential or anticipated safety weaknesses and threats.
4 In addition to gathering such information as above, the OHSP defines communication channels to ensure that data on potential threats and weaknesses is properly transmitted and shared with all personnel involved in the project.
5 The OHSP defines mechanisms for developing and maintaining the capability to identify future threats to safety, quality, and operational performance.
6 Mechanisms are defined to ensure that identified threats and opportunities are conveyed or reflected in future Health and Safety Plans.

USE THE BOX BELOW TO MAKE ANY COMMENTS YOU FEEL APPROPRIATE ABOUT THE OHSP. For example, you can comment on: What are the main barriers or difficulties in drawing up the Occupational Health and Safety Plans? What aspects of the Occupational Health and Safety Plans should be improved?
